# Genomic Analyses of Human European Diversity at the Southwestern Edge: Isolation, African Influence and Disease Associations in the Canary Islands

**DOI:** 10.1093/molbev/msy190

**Published:** 2018-10-05

**Authors:** Beatriz Guillen-Guio, Jose M Lorenzo-Salazar, Rafaela González-Montelongo, Ana Díaz-de Usera, Itahisa Marcelino-Rodríguez, Almudena Corrales, Antonio Cabrera de León, Santos Alonso, Carlos Flores

**Affiliations:** 1 Research Unit, Hospital Universitario N.S. de Candelaria, Universidad de La Laguna, Santa Cruz de Tenerife, Spain; 2 Genomics Division, Instituto Tecnológico y de Energías Renovables (ITER), Santa Cruz de Tenerife, Spain; 3 CIBER de Enfermedades Respiratorias, Instituto de Salud Carlos III, Madrid, Spain; 4 Department of Genetics, Physical Anthropology and Animal Physiology, University of the Basque Country UPV/EHU, Leioa, Bizkaia, Spain

**Keywords:** consanguinity, ROH, local ancestry, MHC, natural selection

## Abstract

Despite the genetic resemblance of Canary Islanders to other southern European populations, their geographical isolation and the historical admixture of aborigines (from North Africa) with sub-Saharan Africans and Europeans have shaped a distinctive genetic makeup that likely affects disease susceptibility and health disparities. Based on single nucleotide polymorphism array data and whole genome sequencing (30×), we inferred that the last African admixture took place ∼14 generations ago and estimated that up to 34% of the Canary Islander genome is of recent African descent. The length of regions in homozygosis and the ancestry-related mosaic organization of the Canary Islander genome support the view that isolation has been strongest on the two smallest islands. Furthermore, several genomic regions showed significant and large deviations in African or European ancestry and were significantly enriched in genes involved in prevalent diseases in this community, such as diabetes, asthma, and allergy. The most prominent of these regions were located near *LCT* and the HLA, two well-known targets of selection, at which 40‒50% of the Canarian genome is of recent African descent according to our estimates. Putative selective signals were also identified in these regions near the *SLC6A11-SLC6A1*, *KCNMB2*, and *PCDH20*-*PCDH9* genes. Taken together, our findings provide solid evidence of a significant recent African admixture, population isolation, and adaptation in this part of Europe, with the favoring of African alleles in some chromosome regions. These findings may have medical implications for populations of recent African ancestry.

## Introduction

The Canarian Archipelago consists of seven main islands located in the Atlantic Ocean ∼100 km from the northwest African coast. By no later than 2,500 years B.P. (Onrubia Pintado 1987) and until the XVth century, when the Spanish conquest began, the Canary Islands were inhabited by the indigenous Guanche population (Crosby 1999). Many anthropological, archaeological, and cultural traits indicate that the most likely origin of Guanche aborigines was the Berber population from North Africa (Hooton 1970), and supporting evidence indicates more than one aboriginal settlement from the Maghreb and the Sahara likely occurred (Arco and Navarro 1987). The Spanish conquest can be divided into two stages: 1) occupation of the less populated islands, which was concluded rapidly and peacefully, and 2) a subsequent slower and violent invasion of the more populated islands, which ended in 1496 (de Abreu Galindo and Cioranescu 1977). Despite the devastating effect of the conquest, many aborigines remained in the territory, either freed or enslaved. The strategic location of the Canary Islands (located between the Americas and Africa) stimulated a continuous immigration between the XVIth and XIXth centuries from Europe and sub-Saharan Africa, with immigration from the latter occurring as a result of the slave trade (Lobo-Cabrera 1993). By the XVIth century, the chronicles estimated the population size as ∼35,000 inhabitants, of which nearly 11,000 were potentially of aboriginal or sub-Saharan African origin (Wölfel 1930). Physical anthropology studies of the inhabitants provided evidence of the continuity of indigenous traits during the XXth century (Falkenburger 1942; Ara 1959; Berthelot 1978).

The aboriginal, historical, and contemporary populations inhabiting the Canary Islands have been the subjects of many population-genetics studies. Among these studies, those focusing on uniparental inheritance markers in samples from current inhabitants (Rando et al. 1999; Flores et al. 2003) and ancient DNA studies of aboriginal (Maca-Meyer et al. 2004; Fregel et al. 2009; Rodríguez-Varela et al. 2017) and historical remains (Maca-Meyer et al. 2005) have yielded consistent findings. Overall, the genetic evidence strongly supports a nearby North African origin of Guanches. Although this ancestry is still evident in present-day inhabitants of the archipelago, other genetic influences from Europe and Africa are also apparent. These other genetic influences have been explained by the shifts in ethnic mixtures after the Spanish conquest. However, because of the sexual asymmetry in the genetic contributions of the ancestral populations (Flores et al. 2001), autosomal markers offer more unbiased estimates than uniparental markers of the recent admixture of European (EUR), North African (NAF) and sub-Saharan African (SSA) ancestry among present-day Canary Islanders. Classical studies analyzing a few blood groups, red-blood-cell enzymes, or approximately a dozen polymorphic *Alu* insertions have shown that this three-way admixture model encompasses a prominent EUR ancestry (62–78%) and as much as 20–38% NAF and 3–10% SSA ancestries (Flores et al. 2001; Maca-Meyer et al. 2004). Studies using single nucleotide polymorphisms (SNPs), although limited by number of genetic markers (Pino-Yanes et al. 2011) or sample size (Botigué et al. 2013), have shown agreement in placing the population ancestries at ∼75–83% EUR, <2% SSA, and as much as 17–23% NAF. Therefore, although NAF ancestry is widespread in Southern Europe (particularly in southwestern populations), these estimates suggest that NAF ancestry in Canary Islands populations reaches the highest levels so far described for Europe (Botigué et al. 2013).

We recently showed that the genetic diversity related to NAF ancestry has important biomedical implications for EUR populations (Botigué et al. 2013). Because of its unique genetic admixture and/or environmental exposures (likely involving evolutionary adaptations), the population inhabiting the Canarian Archipelago suffers from a disproportionate burden of prevalent chronic conditions and associated complications. For example, the prevalence of asthma and allergic diseases in children in the Canary Islands is markedly higher than that in mainland Spain (Sánchez-Lerma et al. 2009). Diabetes, obesity, and hypertension are also more prevalent among Canary Islanders than in other Spanish populations in all age groups (Marcelino-Rodríguez et al. 2016). Moreover, despite the Canarian Archipelago and mainland Spain share the healthcare system, diabetes-related mortality in the Canary Islands remains the highest in the country, and the incidence of diabetes-related morbidities, such as end-stage renal disease and lower limb amputation, differ between the two regions (Aragón-Sánchez et al. 2009; Lorenzo et al. 2010).

Here, we aimed to characterize in detail the genomes of Canary Islanders at an unprecedented scale using SNP arrays and whole genome sequencing (WGS) of samples from all seven islands. We also assessed these data to evidence loci showing large deviations in ancestry with respect to the average of the genome, putative targets of selection and disease associations. In addition, because the population offers a uniquely challenging admixture scenario (i.e., a three-way admixture combined with the admixture of parental populations exhibiting small to moderate degrees of differentiation), this study secondarily evaluated the performance of two of the fastest and most accurate methods of local ancestry estimation for multiway admixtures (Chimusa et al. 2014; Guan 2014).

## Results

### Time since Admixture and Genomic Ancestry Proportions

**Fig. 1.**
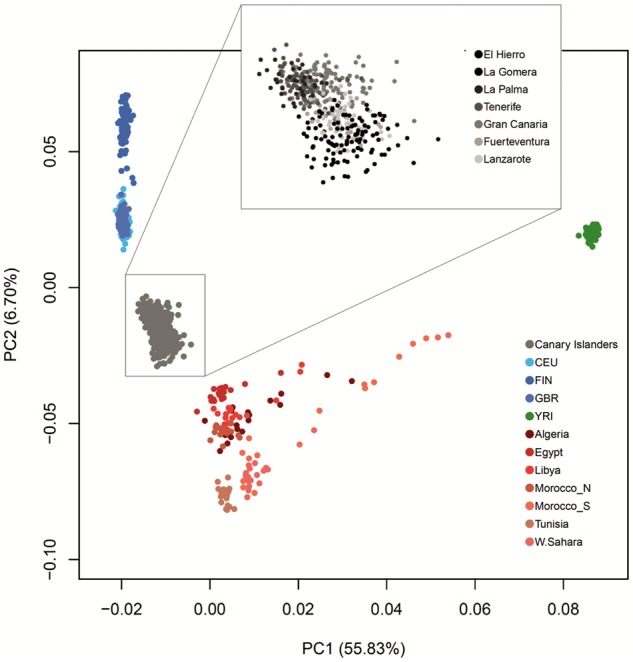
Plot of the first two principal components (explaining 62.5% of variability) from PCA of Canary Islanders and samples from reference populations from Europe, North Africa, and sub-Saharan Africa. The inset depicts a detailed view per island. Results are based on a subsample of 100,175 SNPs excluding those in high LD (pairwise *r*^2^ threshold = 0.5).

After quality control procedures, the intersection with reference data sets, and the exclusion of regions in high-linkage disequilibrium (LD), a total of 100,175 SNPs (*r*^2^ threshold of 0.5) from 416 individuals (34 from El Hierro, 35 from La Palma, 78 from La Gomera, 64 from Tenerife, 117 from Gran Canaria, 32 from Fuerteventura, and 56 from Lanzarote) were used for the analyses. The leading principal components (PCs) (explaining 62.54% of total variation) from the principal component analysis (PCA) including reference populations evidenced the intermediate position of Canary Islanders between the NAF and EUR populations (separated by PC2) and their more distant relationships with SSA, separated by PC1 (fig. 1). In addition, despite the relative homogeneity of ancestry supported by the tight clustering of populations from the different islands, individuals from La Gomera and El Hierro clustered closer to NAF, whereas those from Tenerife and Gran Canaria clustered closer to EUR. These results suggest that there is no clear relationship between the geographic and genetic distances from Africa in the Canary Islands.

We next examined whether Canary Islanders as a whole can be considered an admixed population based on formal tests. To do so, for each pair of reference populations considered as surrogates for the true ancestral populations, we used ALDER to calculate a weighted two-locus admixture LD statistic based on the decay (supplementary fig. 1, Supplementary Material online) and assess whether this statistic supports an admixture of the ancestral populations. In all of the pairs considering one EUR population and one SSA population as proxies of the ancestral populations, there was consistent and significant evidence of admixture (*P* < 3.1 ×10^−69^). Using ALDER, we also estimated the number of generations since the last admixture event. The results were consistent across comparisons and indicated that the admixture event took place ∼13.6 ± 0.7 generations ago (429–495 years BP), which is within the timescale of the historical conquest of the archipelago in the XVth century.

**Fig. 2.**
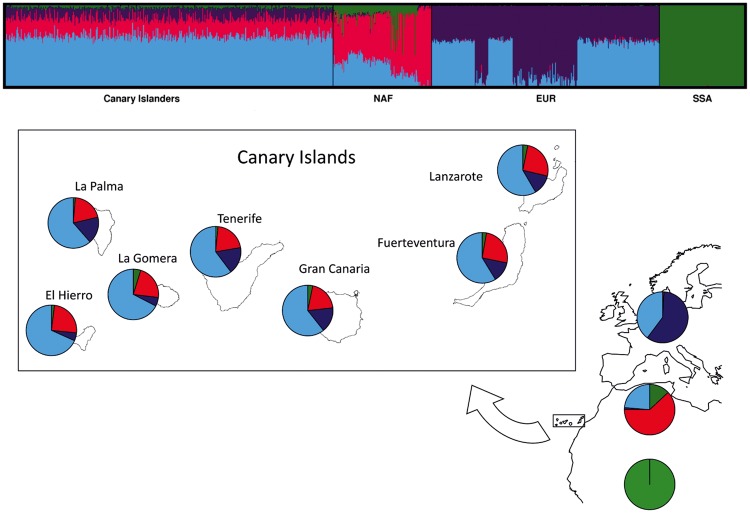
ADMIXTURE estimates for *K* = 4 for Canary Islanders and samples from reference populations from Europe, North Africa, and sub-Saharan Africa.

To complement these results, we examined the ancestry proportions based on a clustering analysis using ADMIXTURE with *K* varying from 2 to 7 (supplementary fig. 2, Supplementary Material online). This revealed a clear ancestry component separating SSA from the other populations from *K* = 2 through *K* = 7 and being absent in EUR individuals. At *K* ≥ 3, a new ancestry cluster became apparent, which reached its maximum frequency in EUR and Canary Islanders. At *K* = 4 and above, ∼40–60% of the ancestry clusters detected in NAF individuals were assigned to a new cluster with its maximum in these populations (>95% on an average in Tunisians). This NAF-related ancestry was also evident in Canary Islanders. Other ancestry clusters emerged for *K* > 4, which generally reflected both further ancestry sharing or unresolved clustering in NAF and Canary Islanders and additional ancestry subdivisions among NAF populations compatible with the mixed pattern of ancestry components evidenced elsewhere (Henn et al. 2012; Arauna et al. 2017). Cross-validation error was lowest for *K* = 4 (fig. 2). At this value, the clusters roughly corresponded to the ancestries, reaching their maximum frequencies in SSA and NAF plus two other ancestry clusters defined for EUR. The results for EUR are largely compatible with previous observations (Seldin et al. 2006). To provide further support to the ancestry clusters evidenced by the ADMIXTURE analysis, we evaluated the fitting of the admixture model with the optimal measures of haplotype sharing between groups using badMIXTURE. For *K* = 4, we observed small residuals with no systematic patterns on the Canary Islanders, whereas larger residuals were concentrated in one of the ancestral components (NAF) (supplementary fig. 3, Supplementary Material online). Therefore, regardless of the genetic heterogeneity resulting from the merging of different North African populations into a single group, the structure of the residuals indicate that the admixture proportions provided by the ADMIXTURE analysis constitute robust inferences of the ancestries in Canary Islanders.

Based on this evidence, we interpreted the EUR-, NAF-, and SSA-related fractions indicated by the ADMIXTURE analysis as the admixture proportions in Canary Islanders. For *K* = 4, the overall ancestry proportions among Canary Islanders were, on an average, 22% NAF and 3% SSA (table 1). These estimates are highly concordant with our previous results obtained under standard settings (Botigué et al. 2013) despite the latter being based on a data set containing SNPs with moderate levels of LD (*r*^2^ threshold set at 0.5). However, in this study, which comprised a larger and more diverse sample of Canary Islanders, we found a wider NAF-related ancestry interval of 14.9–29.9%, and a slightly higher SSA-related ancestry of as much as 9.2% (table 1 and fig. 2). The use of alternative African data sets from The 1000 Genomes Project (1KGP) as SSA representatives in the ADMIXTURE analysis did not qualitatively change the results as the ancestry estimates were highly correlated (for *K* = 4, *R*^2^≥0.995; *P* < 2.2×10^−16^ in all comparisons). Similarly, balancing the number of individuals from the reference populations (*n* = 75 for each) resulted in similar ancestry estimates (averages of 26% NAF and 1% SSA). Overall, these results demonstrate that the Canary Islanders are closely related to EUR but show substantial influences from NAF and distant relatedness with SSA.

**Table 1.** Genomic Ancestry Proportions (from ADMIXTURE, *K* = 4) in Canary Islanders.

	North African			Sub-Saharan African		
	Min.	Mean	Max.	Min.	Mean	Max.
Fuerteventura	0.218	0.255	0.296	0.011	0.027	0.046
Lanzarote	0.214	0.254	0.296	0.014	0.032	0.057
Gran Canaria	0.155	0.200	0.264	0.005	0.032	0.082
Tenerife	0.149	0.208	0.255	0.002	0.015	0.057
La Gomera	0.160	0.221	0.289	0.013	0.048	0.092
La Palma	0.170	0.200	0.245	0.000	0.013	0.032
El Hierro	0.192	0.246	0.299	0.005	0.020	0.032

There was no difference in the NAF-related ancestry between the eastern (Fuerteventura, Lanzarote, and Gran Canaria) and western islands (Tenerife, La Gomera, La Palma, and El Hierro) (22.4% vs. 21.8%, respectively; Wilcoxon test *P* = 0.160), although the SSA-related ancestry differed significantly between the eastern and western islands (3.1% vs. 2.8%, respectively; Wilcoxon test *P* = 6.4×10^−5^). However, the largest differences were observed between the islands that were conquered first and more peacefully by the Spanish (Fuerteventura, Lanzarote, La Gomera, and El Hierro) and those in which the conquest took longer (Tenerife, Gran Canaria, and La Palma) (24.0% vs. 20.3%, respectively, for the NAF-related fraction, and 3.5% vs. 2.4%, respectively, for the SSA-related fraction; Wilcoxon test *P* < 3.0×10^−5^ for both comparisons). This observation agrees with findings based on mitochondrial DNA (mtDNA) lineages that suggest increased African affinities on the former islands (Rando et al. 1999). However, they differ from those based on the nonrecombining portion of the Y chromosome (NRY) that indicate limited NAF affinities of paternal genetic markers on all of the Canary Islands but particularly the populations from the westernmost islands (Flores et al. 2003). Such different distributions of NAF and SSA ancestries, previously evidenced by only uniparental markers, are expected given the sexual asymmetry of parental contributions detected in the current and historical populations of the archipelago (Flores et al. 2001; Fregel et al. 2009).

### Population Isolation

**Fig. 3.**
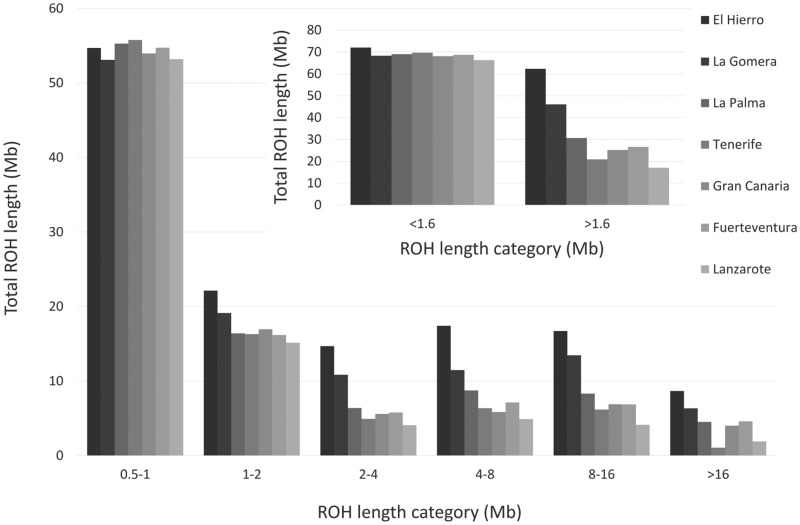
Average length (Mb) of ROHs using two classifications into categories in the populations from the Canary Islands.

Recent studies have indicated that runs of homozygosity (ROHs) are common to all world populations, are longer than expected, and have profiles that can indicate distinctive demographic histories of the population and of inbreeding (Kirin et al. 2010; Pemberton et al. 2012). Here, we have analyzed, for the first time, genome-wide ROH patterns in Canary Islanders to reveal the level of population isolation. Focusing first on the average total sum of ROHs over all subjects sampled, we found that <10 Mb of the Canary Islanders genome on an average was in ROHs of >2 Mb in length. For smaller ROHs (≤1.6 Mb), which have been associated with geographic distances from East Africa (Pemberton et al. 2012), the profiles were very similar across the island populations (fig. 3). However, among all ROHs >1.6 Mb, the samples from La Gomera and El Hierro showed consistently higher average total ROHs than did the samples from the remaining islands, suggestive of increased recent inbreeding in these two islands.

**Fig. 4.**
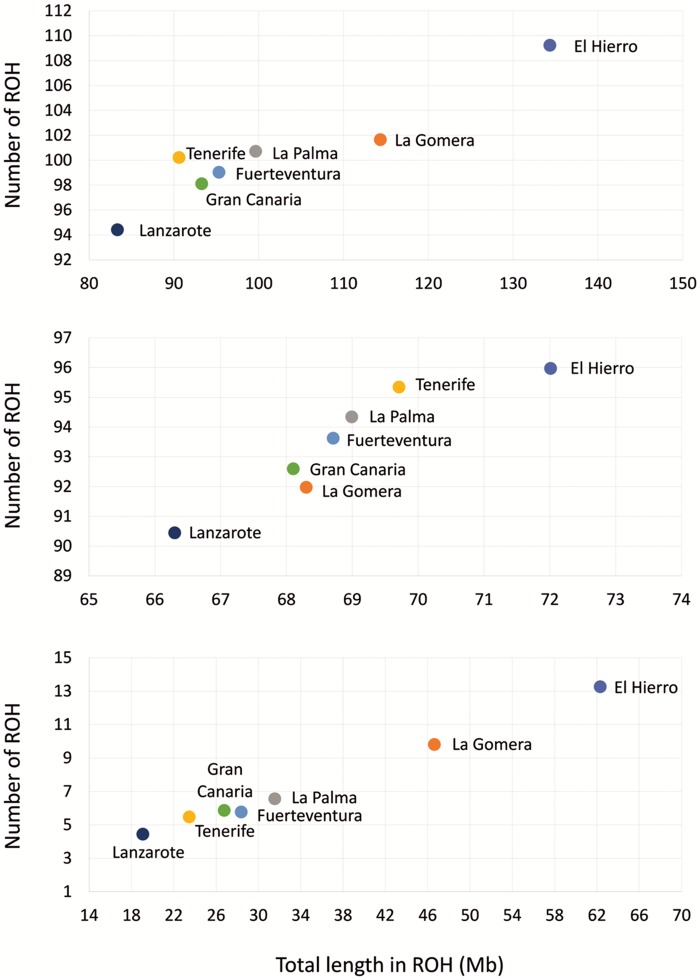
Average number of genome regions in ROHs with respect to the average total (top), ≤1.6 Mb (middle), and >1.6 Mb (bottom) lengths of ROHs per island.

An exploration of average total ROHs by island again indicated El Hierro and La Gomera as population outliers. The genomes from these two islands showed the largest number of fragments in ROHs and the longest average total ROH length (fig. 4), the latter demonstrating significant differences from all the other islands (Wilcoxon test *P* < 1.0×10^−3^ for all pairwise comparisons) except La Palma. Interestingly, La Gomera and El Hierro showed average total ROH lengths of 114 and 134 Mb. The conclusions remained unchanged when considering only ROHs >1.6 Mb for the average total ROHs estimates (Wilcoxon test *P* < 1.0×10^−3^ for all pairwise comparisons), whereas the differences disappeared when using the average total of ROHs ≤1.6 Mb (lowest *P* = 4.5×10^−3^). Given that variant ascertainment of the array is less of a problem for ROHs >1Mb (Ceballos et al. 2018), we interpret the ROH patterns observed for La Gomera and El Hierro as signatures of genetic isolation, reduced population size and consequently, endogamy within the archipelago. In this respect, although the ROH patterns suggested a tendency toward larger average total ROH length in the western and smaller islands of the archipelago, the mean number of genomic regions in ROHs significantly increased with geographical longitude (Spearman’s rank correlation rho = 0.93, *P* = 6.7×10^−3^).

### Local Ancestry: Assessing Estimators and the Average Size of Blocks

**Fig. 5.**
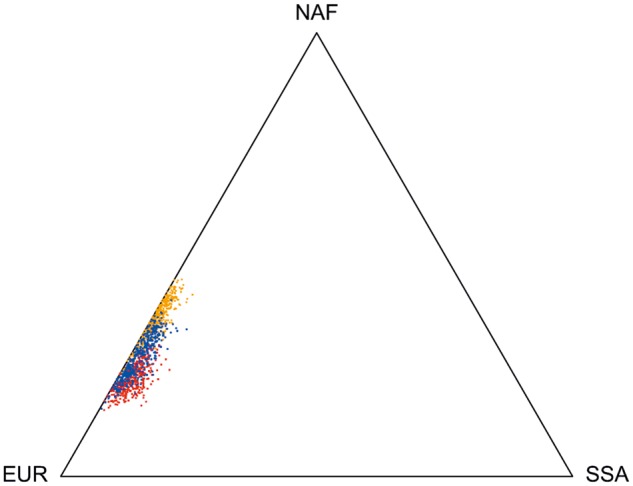
Triangle plot of individual genomic admixture proportions in Canary Islanders as estimated by ADMIXTURE with *K* = 4 (red), ELAI (blue), and LAMP-LD (orange).

**Fig. 6.**
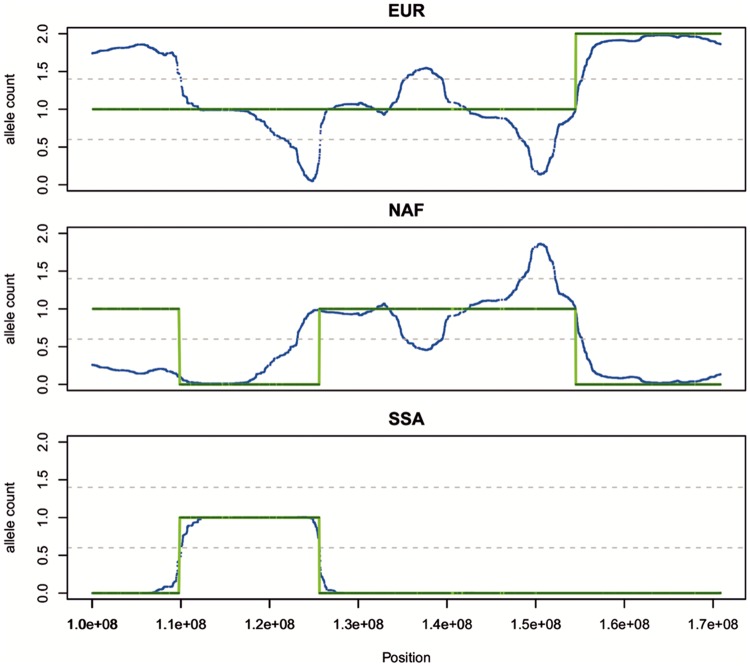
Inference of local ancestry by ELAI and LAMP-LD. The plot shows an example of inference in a chromosome region (one panel of each parental population) comparing ELAI (blue) with LAMP-LD (green) allele dosages.

In cases of recent admixture, the genomes of admixed individuals become a mosaic of chromosomal stretches originating from the ancestral populations, which can be detected by examining locus-specific ancestry (usually termed local ancestry). Many local ancestry methods perform well in two-way admixture scenarios with large genetic differences between the ancestral populations (e.g., in African–Americans). However, the difficulty increases with the number of ancestral populations (e.g., three-way admixtures), particularly if a small to moderate degree of differentiation exists between some of the ancestral populations (e.g., between NAF and EUR). Given that this study constitutes the first time that the patterns of ancestry blocks in Canary Islander genomes have been assessed, we first evaluated two of the fastest and most accurate methods of local ancestry estimation for multiway admixtures, ELAI and LAMP-LD, both of which have been fruitfully utilized with Hispanic populations (Baran et al. 2012; Zhou et al. 2016). We compared their results of these methods with those obtained previously by ADMIXTURE, pooling the two EUR-related ancestry fractions for the comparisons. The Pearson correlations between the estimates of ADMIXTURE and either ELAI or LAMP-LD were strongly significant (*P* < 5.0×10^−10^ in all cases) (table 2). However, ELAI offered better fitting estimates than LAMP-LD based on the overall estimates of NAF- and SSA-related ancestries obtained. Whereas the ELAI estimates were similar to those of ADMIXTURE (estimates for NAF and SSA of 23.6% and 2.3%, respectively), LAMP-LD provided an inflated estimate of NAF-related ancestry (estimates for NAF and SSA of 32.7 and 1.4%, respectively) (fig. 5). A least squares estimator indicated that individual fractions were between 2 (SSA) and 4 times (EUR and NAF) as different between ADMIXTURE and LAMP-LD than between ADMIXTURE and ELAI (not shown). In addition, whereas the lengths of the ancestry blocks provided by ELAI and LAMP-LD were correlated for the three ancestries (fig. 6), ELAI provided greater sensitivity for the detection of smaller stretches of ancestry. Based on these results, all further local ancestry analyses were conducted with ELAI estimates.

**Table 2.** Pearson Correlation between ADMIXTURE (*K* = 4) and ELAI or LAMP-LD Estimates of Ancestry in Canary Islanders.

	ELAI		LAMP-LD	
Ancestry	Coef.	95% CI	Coef.	95% CI
European	0.95	0.94–0.96	0.83	0.80–0.86
North African	0.88	0.85–0.90	0.74	0.68–0.78
Sub-Saharan African	0.93	0.92–0.94	0.89	0.87–0.91

Block sizes followed lognormal distributions (supplementary fig. 4, Supplementary Material online), with the largest blocks on an average corresponding to the EUR-related component (13.05 Mb) followed by the NAF (8.46 Mb) and SSA (7.48 Mb) components, and with all pairwise comparisons yielding significant differences (Wilcoxon test *P* < 3.0×10^−8^). Based on these block length estimates and the ADMIXTURE proportions calculated for all Canary Islanders, we would expect averages of ∼181 EUR-, 82 NAF-, and 13 SSA-related blocks per haploid genome (i.e., 276 ancestry blocks in total), which are lower than but within the range of the estimates previously suggested for African–Americans (Shriner et al. 2011).

### Deviations in Local Ancestry and Selection

**Fig. 7.**
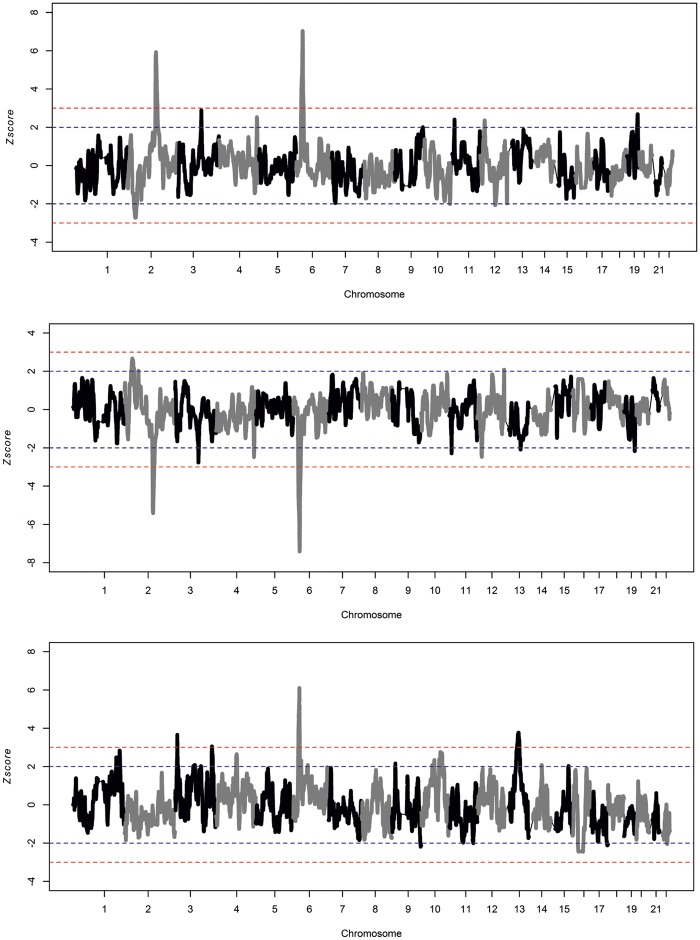
Genome-wide *Z*-score scan of ELAI local admixtures in Canary Islanders for NAF (top), EUR (middle), and SSA (bottom). Horizontal broken lines (blue > |2|; red > |3|) indicate score thresholds.

Based on the previous results, we then used the ELAI estimates to assess the existence of regional deviations in any of the three ancestries in Canary Islanders. We detected eight peaks with large ancestry deviations: two enriched in EUR ancestry, and six associated with higher proportions of African ancestries (fig. 7, table 3, and supplementary fig. 5, Supplementary Material online). Notably, the EUR- and NAF-related peak positions largely overlapped. The EUR-related peaks were located on chr2 (with the lead SNP showing an average local ancestry of 55% and flanked by the *CXCR4* and *THSD7B* genes) and chr6 (with all lead SNPs located within *HLA-B*, with an average local ancestry of 49%). The two peaks of NAF-related ancestry enrichment were located on chr2 (with the lead SNP showing an average local ancestry of 43.2% and flanked by the same genes flanking the EUR-related peaks) and chr6 (with the lead SNP showing an average local ancestry of 46.3% and flanked by the *NFKBIL1* and *LTA* genes). Peaks associated with SSA-related ancestry were situated on chr3 (two hits: one with the lead SNP located within *SLC6A11*, the other with the lead SNP located within *KCNMB2*, both presenting an average local ancestry of ∼4.0%), chr6 (with the lead SNP within *ZNRD1*, showing an average local ancestry of 5.9%), and chr13 (with the lead SNP near *PCDH9* and flanked by two long intergenic noncoding RNA genes: *LINC00355* and *LINC01052*, showing an average local ancestry of 4.4%). These regions span several Mbs (range of 1.2–12.3 Mb), meaning that, while the observed ancestry deviations may indicate adaptive processes, any adaptation may be unrelated to the gene harboring the lead SNP. However, it is likely that the *LCT* and HLA loci caused the peaks on chr2 and chr6. One regulatory variant of *LCT* is the major determinant for lactase persistence in EUR populations and underlies the strongest signal of selection in the human genome identified to date (Mathieson et al. 2015). Similarly, this region has been shown to be under positive selection in Bantu-speaking populations (Patin et al. 2017). As for HLA, the gene-rich human leukocyte antigen region is also a well-known target of selection in EUR and African populations, likely involving balancing selection (Gurdasani et al. 2015; Mathieson et al. 2015; Patin et al. 2017) as well as directional selective processes, such as that described for *HLA-B* alleles and malaria protection in the Sahel (Sanchez-Mazas et al. 2017). Based on this evidence, we reasoned that all or some of the remaining regions with ancestry deviations detected (two on chr3 and one on chr13) could also have resulted from selective processes. One of the hallmarks of selection is increased homozygosity and reduced diversity in the surrounding regions (Pickrell et al. 2009). Therefore, we then assessed the mean heterozygosity and the number of samples with ROHs containing SNPs from the ancestry-enriched regions. We performed this assessment for all peaks associated with African ancestry (including those in chr2 and chr6) to obtain references for comparisons (table 4) and found that one of the regions on chr3 and the region on chr13 showed signals of reduced diversity among Canary Islanders. To formally test for the existence of selective signals in chr3 and chr13 loci, we assessed the Tajima’s *D* and the Population Branch Statistic (PBS) in a subset of individuals for which WGS data was available. Deviations from neutrality at three locations were revealed by at least one test for the three loci, with frequent support for more than one hit obtained for some regions after accounting for the number of comparisons (*P* < 0.017 and *P* < 0.01 for Tajima’s *D* and PBS, respectively, after Bonferroni correction). These three locations were as follows: in the intergenic region between the *SLC6A11* and *SLC6A1* genes on chr3 (Tajima’s *D*=−1.733, *P* < 0.061; PBS = 0.108, *P* < 2.0×10^−4^), near *KCNMB2* on the same chromosome (Tajima’s *D*=−1.511, *P* < 0.130; PBS = 0.064, *P* < 2.0×10^−4^), and in the intergenic region between *PCDH20* and *PCDH9* on chr13 (Tajima’s *D*=−2.505, PBS = 0.136, *P* < 2.0×10^−4^ in both comparisons) (table 5). Interestingly, common genetic variants previously found to be associated with height (He et al. 2015), bone traits (Kiel et al. 2007), and asthma (Ferreira et al. 2011) reside in two of these regions (table 5). This observation is in agreement with the observation that variants for inflammatory diseases located via genome-wide association studies are significantly enriched in signatures of positive selection in European populations (Raj et al. 2013).

**Table 3.** Genomic Regions with Supported Deviations in Ancestry among Canary Islanders.

Ancestry	Region	Lead SNP	*Z* Score	Mean Ancestry
North African	chr2: 133,952,040-144,266,489	rs10177911	5.92	43.2
	chr6: 24,703,442-36,288,651	rs2844484	7.03	46.3
European	chr2: 134,088,150-142,882,593	rs4954402	−5.41	55.0
	chr6: 24,120,456-36,653,597	(*)	−7.41	49.0
Sub–Saharan African	chr3: 10,539,482-11,710,471	rs17033567	3.65	4.3
	chr3: 177,443,968-178,679,751	rs13061192	3.02	3.9
	chr6: 24,790,462-32,192,083	rs16896944	6.10	5.9
	chr13: 57,962,413-70,091,195	rs9540226	3.77	4.4

(*) rs2524095, rs16899203, rs16899205, rs16899207, rs2524089, rs2394967, rs2524066, rs9366778.

**Table 4.** Diversity Estimates in Regions with Large Deviations in African Ancestry in Canary Islanders.

	Heterozygosity		SNPs in ROHs	
Region	Mean	*P* value^a^	Mean	*P* value^a^
Genome	0.300	–	12.508	–
chr2: 133,952,040..144,266,489	0.298	0.452	11.934	1.38×10^−7b^
chr3: 10,539,482..11,710,471	0.278	0.010	9.155	0.037
chr3: 177,443,968..178,679,751	0.309	0.418	14.172	2.20×10^−16b^
chr6: 24,703,442..36,288,651	0.296	0.015	25.934	2.20×10^−16b^
chr6: 24,790,462..32,192,083	0.285	5.28×10^−7b^	29.364	2.20×10^−16b^
chr13: 57,962,413..70,091,195	0.287	2.55×10^−4b^	22.050	2.20×10^−16b^

a Wilcoxon rank sum test.

b Statistically significant after adjusting for multiple tests (*P* < 8×10^−3^).

**Table 5.** Tajima’s *D* and PBS Test Results Calculated for chr3 and chr13 Regions with a *Z* score>|3|.

	Tajima’s *D*			PBS		
Chr.	Region (×1,000)	Score (probability)	Gene	Region (×1,000)	Score (*P* < 2.0×10^−4^)	Gene (traits)
3p25.3	11,010–11,020	−1.733 (0.061)	*SLC6A11*-*SLC6A1*	11,025–11,035 11.635–11.645	0.108 0.087	SLC6A1 *VGLL4* (height^a^)
3q26.32	178,120–178,130	−1.510 (0.130)	*KCNMB2*	177,465–177,475 178,573–178,583	0.177 0.064	LINC00578 KCNMB2
13q21.32	66,060–66,070	−2.505 (<2.0×10^−4^)	*PCDH20*-*PCDH9*	63,600–63,650	0.136	*PCDH20*-*PCDH9* (bone traits^b^, asthma^c^)

a He et al. (2015).

b Kiel et al. (2007).

c Ferreira et al. (2011).

One striking observation relates to the chr2 peak, which indicates a higher proportion of NAF-related alleles in Canary Islanders in this region. Given that at least three other *LCT* variants have been linked with lactase persistence in other populations, we then explored whether any of those variants existed in this population and could offer a potential explanation for this peak. By accessing the WGS data available for a subset of 14 subjects, we found that the only detected variant position associated with lactase persistence was rs4988235 (a.k.a. -13,910), which is the major determinant for lactase persistence in Europe. The frequency of this lactase persistence allele in Canary Islanders (-13,910*T, 40%) is intermediate between the frequency reported in other central and northern EUR populations (60–80%) and that in NAF populations (24%) (Bersaglieri et al. 2004; Ben Halima et al. 2017) (http://www.ucl.ac.uk/mace-lab/resources/glad). Therefore, we suggest that the prevalence of lactase nonpersistence alleles in Canary Islanders and NAF populations likely explains the chr2 peak in Canary Islanders. In fact, a recent ancient DNA study of pre-Hispanic human teeth from a small sample of five Guanche people from Tenerife and Gran Canaria also suggested that the dominant phenotype was lactose intolerance (Rodríguez-Varela et al. 2017). Taken together, this evidence reduces the possibility that the known African or Arabian *LCT* variants (Ingram et al. 2009; Ranciaro et al. 2014) are responsible for the chr2 peak, although we cannot rule out the possibility that there may be other rare variants associated with lactase persistence in this population that remain undiscovered.

### Links between Ancestry, Diseases, and Biological Pathways

**Fig. 8.**
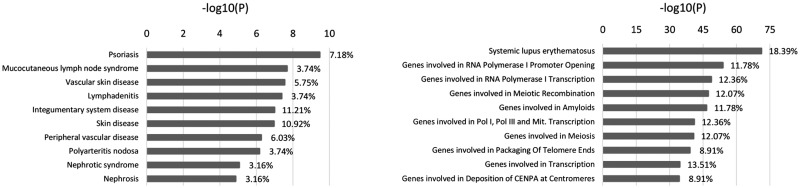
Enrichment analysis on regions with large deviations for any ancestry. Top ten significantly enriched human diseases (left) and MSigDB pathways (right).

To determine whether the genomic regions with large deviations in ancestry are linked with human diseases and biological pathways, we applied enrichment analysis to the 341 unique genes mapping to the regions with significant evidence of EUR-, NAF-, or SSA-related ancestry deviations (fig. 8 and supplementary table 1, Supplementary Material online). The top annotations were dominated by skin, vascular, renal, autoimmune, and neuropsychiatric diseases as well as by DNA metabolism, amyloids, meiosis, and transcription pathways. In addition, many prevalent diseases, such as diabetes, asthma, and allergy, and infectious diseases as well as some severe conditions, such as oncologic and severe acute respiratory syndrome, were also significantly enriched (*q* value < 0.05). Regulation of inflammatory response, the complement pathway, telomere maintenance, and antigen processing and presentation were among the pathways significantly enriched (*q* value < 0.05) but ranked lower in the results (supplementary tables 2 and 3, Supplementary Material online).

## Discussion

The recent history of the Canary Islands has involved heterogeneous genetic influences from Europe and Africa since their aboriginal settlement from nearby North Africa during the first millennium BC. Here, we report new high-density genotyping and WGS data that allowed an exhaustive exploration of the time since the last admixture event, genetic diversity and isolation, disease links, and putative selective processes in these populations. By using the largest and most diverse Canary Islands sample analyzed to date at a genomic scale, we recognized a wider range of interindividual African ancestry assignments (as much as 29.9% NAF and 9.2% SSA), the largest so far identified in southwestern EUR populations (Botigué et al. 2013). Furthermore, a between-islands pattern of variation in African ancestries was observed and interpreted according to the impact of the Spanish conquest and settlement of the territory during and after the XVth century. For the first time, we have found genomic signals of inbreeding in the population, suggesting that isolation has been especially drastic in the two smallest islands, El Hierro and La Gomera, the latter of which is associated with the highest frequency reported to date of the Northwest African U6 mtDNA lineage (>36%) in a non-African population (Rando et al. 1999). In addition, we uncovered the mosaic nature of Canary Islander genomes, finding that a typical haploid genome would consist of fewer than 300 ancestry blocks and the number of EUR-related blocks would be approximately twice and 10 times that of the NAF- and SSA-related blocks, respectively. Finally, we used this information to focus on particular chromosome regions that showed an overrepresentation of one of the recognized ancestries. These regions included the *LCT* gene, the HLA, and other genes that appear to have undergone selective processes. Most importantly, chromosome regions with large deviations in the ancestries were enriched in genes underlying important diseases that disproportionately affect the archipelago, including respiratory diseases and diabetes, among others.

Despite the profound impact of the European immigration starting in the XVth century, our results indicate that significant genetic footprints of African ancestry persist in the current inhabitants of the Canary Islands. Our data also imply that gene flow between the island populations must have been high to maintain the relative homogeneity observed for the African ancestries, despite the significant discontinuity favoring African affinities in the populations of El Hierro, La Gomera, Fuerteventura, and Lanzarote. Strikingly, such a pattern was also revealed in mtDNA studies of independent samples (Rando et al. 1999), suggesting that African ancestry has been better preserved in the populations that were conquered at the beginning of the historic process, where the literature attests to a more peaceful occupation of those territories (Suárez et al. 1988). In contrast to our previous study, which was limited by the number of autosomal markers utilized at the time (Pino-Yanes et al. 2011), in the present study, we were able to measure the existence of a minor but substantial SSA-related influence in the populations of the seven islands. SSA migrations into NAF populations are well known (Rando et al. 1998; Wright 2007), suggesting that continuous gene flow started before the aboriginal settlement of the islands. In addition, our results evidenced significant differences in the mean ancestry block lengths interpreted as of recent SSA and NAF origin in Canary Islanders. While this result may be indicative of admixture events involving different African components, as has been supported by many previous studies (Arco and Navarro 1987; de Castro 1987; Navarro Mederos 1997; Flores et al. 2003), independent analyses should aim to establish the time periods of such events. For example, in a recent genomic study of ancient DNA, a very low proportion of SSA ancestry was observed in pre-Hispanic human remains despite their genetic resemblance to other modern NAF populations (Rodríguez-Varela et al. 2017). This finding suggests that the proportion of SSA ancestry we observed in Canary Islanders likely originated in the postconquest importation of enslaved African people.

Previous genetic studies of pre-Hispanic remains, burial remains from the XVIIth century, and samples from current inhabitants indicate declining frequencies of the NRY lineages ascribed to aboriginal people (Fregel et al. 2009). However, a continuity of aboriginal mtDNA lineages, possibly evolving locally into Canarian-specific U6b subhaplogroups (Rando et al. 1999; Maca-Meyer et al. 2004), has been evidenced in the aboriginal remains and current inhabitants, suggesting an important sexual asymmetry involved in the demographic process of the population (Flores et al. 2001). In this study, we found that five other regions in the autosomal genome, including the HLA and *LCT*, paralleling this scenario, where genetic affinities with African populations remain regionally preserved today. Independent studies and our own data support that putative selective processes are the cause of such autosomal affinities. However, given that the bulk of human adaptation occurs via selection on standing variation (Schrider and Kern 2017), it is possible that these signals are not due to private alleles but to preexisting variations in any of the parental populations involved in the admixture. Indeed, extensive evidence indicates pervasive selection processes in the HLA and *LCT* regions in different populations; these processes have been explained by dietary and cultural changes occurring with the Neolithic dispersion, including population density increases and the establishment of permanent settlements, resulting in novel pathogen exposures (Marciniak and Perry 2017).

Additionally, the *SLC6A11* and *KCNMB2* genes have been suggested to be under selective pressures in African and American populations (Hu 2012; Watkins et al. 2012), and literature evidence supports *DIAPH3*, not *PCDH9*, as the closest candidate target for selection in the chr13 region (Pickrell et al. 2009). One important consequence of this observation is that those regions contain genes in which mutations are linked to rare disorders that are usually screened in families with genetic risks, such as *PCDH9*, *SLC6A11*, *SLC6A1*, and *COL11A2*. Therefore, as local ancestry will act as a confounder in the context of genetic tests, disease genes linked to those regions could be candidates for interpretative problems in diagnosis (Manrai et al. 2016). However, beyond monogenic conditions, such problems can arise in the context of complex disease mapping and in the evaluation of known risk factors. The HLA region, a central locus in the predisposition for or protection from many diseases (Hill et al. 1991; Oksenberg et al. 2004; Pelak et al. 2010), contains genetic markers that have been previously identified as related to complex traits such as asthma susceptibility (*MUC22*) (Galanter et al. 2014) and HIV-1 infection control (*HLA-C*) (Thomas et al. 2009), and their attributable risks would have passed unnoticed if local ancestry was not appropriately accounted for in the studies. Furthermore, the regions exhibiting local ancestry deviations have significant associations with distinct prevalent diseases in this population. Therefore, it can be speculated that the prevalence of some of these diseases in the Canary Islands population might be influenced by the distinctive genetic makeup of this population. This hypothesis is in agreement with epidemiological studies of cardiovascular risk factors (Cabrera de León et al. 2006; Bueno et al. 2008; Marcelino-Rodríguez et al. 2016), asthma and allergies (Sánchez-Lerma et al. 2009; Juliá-Serdá et al. 2011).

To the best of our knowledge, this is the first study to evaluate the proportion of the genome in ROHs in this population. Our results support distinct degrees of isolation and consanguinity between the seven islands, which are most extreme in the two smallest islands, La Gomera and El Hierro. Because of isolation, the inhabitants of these islands would be enriched in low-frequency functional variants (Xue et al. 2017) that can lead to novel discoveries of disease genes (Moltke et al. 2014; Jakobsdottir et al. 2016). As a consequence, there is an increased number of recessive variants that can confer risk for complex diseases (Rudan et al. 2003; Campbell et al. 2007; Lencz et al. 2007; Ghani et al. 2015; Thomsen et al. 2016). In addition, founder monogenic mutations are expected, as observed in distinct Canarian populations for type 1 primary hyperoxaluria (Santana et al. 2003; Lorenzo et al. 2006, 2014), sickle-cell anemia (Castella et al. 2011), Wilson’s disease (García-Villarreal et al. 2000), and cardiovascular traits (Rodríguez-Esparragón et al. 2017), highlighting the singular genetic characteristics of Canary Islanders.

It is important to declare some limitations of our study. First, scarce genomic data are available for NAF populations in public databases (Henn et al. 2012). As a consequence, there was limited overlap in SNP contents with the SNP array utilized, leaving us with as few as 114,567 SNPs for some components of the study. This circumstance forced us to use 1KGP population references to maximize SNP density in the comparisons. Along with the number of generations since admixture and the ascertained nature of the contents of the array, these conditions likely had direct impacts on the local ancestry estimates, the average lengths and the regions identified by the admixture scan. However, further studies will aim to improve this overlap by further WGS and/or SNP array genotyping of novel NAF samples to be able to refine future scans. The regions identified by the admixture scan are relatively wide, on the order of several Mb, which limits the precise allocation of ancestry peaks. Therefore, the genes highlighted by proximity to the detected peaks should be considered with caution. In this respect, the final data set had low or no coverage of centrosomes, offering no basis for local ancestry inference in those regions. Although this situation would have affected all ancestries equally, we excluded those regions from the analysis to avoid an upward bias in the average block length measures. Furthermore, the choice of reference populations for ancestry inference is a common concern in these studies. In our case, we balanced choosing a reference data set with sufficient genetic resolution to retain the minimal number of SNPs required for the analyses (>100K) with the use of EUR or SSA populations where NAF or Near East influences were absent or minimal, as both factors hamper local NAF ancestry inferences. In any case, a ubiquitous Berber substrate was recently found in both pre-Hispanic remains from the aboriginal Guanche people and samples from modern NAF populations, supporting a close genetic affinity between these populations (Rodríguez-Varela et al. 2017). Finally, even in the ideal scenario of having access to population data sets from all over the world, most natural populations are expected to represent heterogeneous groups as well. This heterogeneity was the justification for considering all available NAF data sets as a single population source in the model. However, we admit that the reference population sources utilized in the study constitute model simplifications.

In conclusion, here we have provided a genetic dissection of population ancestries and isolation in Canary Islanders at an unprecedented level. We estimate that up to 34% of Canarian genomes are of recent African descent and that the geographical distribution of ancestries still reflects historical events. Local ancestry estimates enabled the identification of Mb-size chromosome regions with higher-than-expected African affinities, most likely involving putative adaptive signals. Our results suggest that these observations may have implications for the major health disparities affecting the population. In addition, genetic testing and genetic mapping studies of diseases in Canary Islanders should take local ancestry into account. Finally, because the adaptive signals were previously described in populations from Africa and America, our conclusions could also have repercussions for the identification of disease loci in other recently admixed populations.

## Materials and Methods

### Samples, Genotyping, and Reference Population Data Sets

The sample of Canary Islanders consisted of 429 unrelated subjects who self-declared as having two generations of ancestors born on the same island of the Archipelago. Samples were selected from a large cohort study entitled “CDC of the Canary Islands” (Cabrera de León et al. 2004), which included ∼7,000 randomly selected representatives of the general Canarian population aged between 18 and 75 years and unbiased for gender. Informed consent and an extensive health survey were obtained from all participants through personal interviews. Genotyping of 587,352 variants was conducted using the Axiom Genome-Wide Human CEU 1 Array (Affymetrix, Santa Clara, CA) with the support of the National Genotyping Center (CeGen), Universidad de Santiago de Compostela Node. Genotyping quality control was performed with R 3.2.2 and PLINK v1.07 (Purcell et al. 2007). Thus, samples with genotype call rates <95%, discordant sex and family relationships (PIHAT > 0.2) were removed from the study, leaving a total of 416 individuals, 205 men and 211 women, for further analyses (34 from El Hierro, 35 from La Palma, 78 from La Gomera, 64 from Tenerife, 117 from Gran Canaria, 32 from Fuerteventura, and 56 from Lanzarote). Additionally, those SNPs with a genotyping rate <95%, a minor allele frequency (MAF) <0.01, or deviating from Hardy–Weinberg expectations (*P* < 1.0×10^−6^) were excluded, leaving a total of 516,348 SNPs. To maximize SNP density in the downstream analyses, we relied on the 1KGP Phase 3 data to extract the data sets serving as EUR and SSA sources (Sudmant et al. 2015). According to recent genome-wide ancestry estimates (Botigué et al. 2013), the presence of Near East or NAF influences in EUR and SSA populations is minimal. In addition, NAF ancestry in EUR populations is clearly distinguishable from Near Eastern influences (Botigué et al. 2013). This information motivated the selection of British (GBR) and Finnish (FIN) people and Utah residents with NW EUR ancestry (CEU) (overall *n* = 289) as well as Yoruba Nigerian (YRI) people (*n* = 108) as the representatives for EUR and SSA sources, respectively. To perform sensitivity analyses of the results, random subsamples of 75 individuals from each set of EUR, NAF, and SSA samples were alternatively used as well as other data sets from 1KGP, namely the Gambian Mandenka (GWD; *n* = 113) and Sierra Leone Mende (MSL; *n* = 85) data sets. The NAF representative grouping (*n* = 125) was gathered from samples with origins in North and South Morocco, Occidental Sahara, Algeria, Tunisia, Egypt, and Libya that had previously been genotyped with the Genome-Wide Human SNP Array 6.0 (Affymetrix) (Henn et al. 2012). Using PLINK, we ensured that all samples in the reference data had a genotyping call rate >95% and excluded SNPs with a > 5% missing rate or with a Hardy–Weinberg equilibrium *P* < 1×10^−6^ in at least one population. The intersection of data sets and postfiltering (of SNPs located in mtDNA or the sex chromosomes) left a total of 114,567 SNPs for downstream analyses (data available in http://www.iter.es/wp-content/uploads/2018/09/AffyCEU1_data_from_Canary_Islanders_MBE-Guillen-Guio-et-al.2018.zip).

### Admixture Inference and Population Analyses

Principal Component Analysis (PCA) was performed using PLINK v1.9 (Chang et al. 2015). ALDER v1.03 (Loh et al. 2013) was used to calculate the two-locus decay of admixture LD to test the existence of admixture and to infer the time of the most recent admixture event in Canary Islanders (assuming 33 years per generation). ALDER was first used to pretest all reference populations to determine the best pair of populations to be considered as ancestral for the estimate, avoiding the presence of long-range LD correlations with the admixed population. Then, we were able to test for admixture and date the admixture event using FIN or CEU as surrogates of EUR and YRI, GWD, or MSL as surrogates of SSA. All of the other populations failed in the pretest.

We used ADMIXTURE v1.22 (Alexander et al. 2009), which uses a maximum likelihood estimation of individual ancestries averaged across the genome, to compute the ancestry clusters of each individual and to serve as a reference for assessing the performance of the local ancestry estimators. The ADMIXTURE calculations assumed 2 to 7 ancestral populations (*K*) and used a 10-times cross-validation with random seeds to estimate the best predictive *K*. A subsample of 100,175 SNPs from EUR, NAF, SSA, and Canary Islanders was used for this assessment. This subset resulted from excluding with PLINK those SNPs in LD (window size = 50, step = 10, pairwise *r*^2^ threshold = 0.5) or located in regions of long-range LD according to hg19 positions (chr5: 43,964,243–51,464,244; chr6: 24,892,021–33,392,022; chr8: 7,962,590–11,962,591; and chr11: 45,043,424–57,243,424). The ADMIXTURE results were also assessed for the effects of downsampling the number of samples from the reference populations and the use of alternative SSA surrogates other than YRI (GWD and MSL). To provide further support to the ADMIXTURE results, we assessed the goodness of fit of the ADMIXTURE model to the underlying genomic data based on the patterns of haplotype sharing between individuals using badMIXTURE v0.0.0.9000 (https://github.com/danjlawson/badMIXTURE), whose residuals provide information on the ancestral relationships between the population groups. Statistical differences in ADMIXTURE ancestry estimates between islands and regions were assessed by Wilcoxon test.

To further explore the population history and isolation of Canary Islanders, ROHs were calculated with PLINK based on a previously described sliding window approach (Kirin et al. 2010) considering regions of 5,000 kb (ensuring a minimum density of 50 kb/SNP per window to reduce biases due to differences in local SNP densities), allowing for one heterozygous variant and up to five missing calls per window, and counting those ROHs with a minimum length of 500 kb. As a measure of the average total extent of homozygosity per population, we then calculated the average lengths of the ROHs in six categories (0.5–1, 1–2, 2–4, 4–8, 8–16, and >16 Mb). Additionally, to provide further support to the findings, we classified ROHs according to the size limits suggested by Pemberton et al. (2012) but simply stratified into ROHs ≤1.6 Mb and >1.6 Mb. ROH length patterns in Canary Islanders were also explored by assessing the average number of genome regions in ROHs and by the average total length of ROHs per island. Differences in the average total ROH lengths were assessed by Wilcoxon test adjusting for the number of comparisons via Bonferroni correction (*P* < 2.4×10^−3^ considered significant).

### Local Ancestry Assessments and Block Sizes

Local ancestry blocks across autosomes were inferred by using LAMP-LD v1.0 (Baran et al. 2012) and ELAI v1.0 (Guan 2014) assuming three admixing populations (EUR, NAF, and SSA). These two methods do not require SNPs to be independent and perform well with recent multiway admixtures. However, while ELAI uses multilocus genotype data, overcoming the inherent uncertainty of the phasing step, LAMP-LD requires haplotype data for the parental populations. For that step, we used SHAPEIT v2.727 (Delaneau et al. 2014) for haplotype reconstruction under the default settings. We assumed 15 generations since admixture, which is consistent with our results as well as with assumptions made on studies in populations with similar historic scenarios (Price et al. 2007). We then compared the LAMP-LD and ELAI estimates to those provided by ADMIXTURE by Pearson correlation coefficients and by a least squares estimator of the differences in individual ancestry estimates. Ancestry block sizes were calculated excluding the centromere regions, as they are not adequately covered by genotyping arrays. In addition, given that ELAI provides ancestry dosages in a continuum (0–2), ancestry block size estimates in this case were derived from dosage approximations to the next nonnegative integer estimates (thresholds set at 0.6 and 1.4) (supplementary fig. 6, Supplementary Material online).

### Local Ancestry and Diversity Deviations and Enrichment Analyses

We used the method proposed by Zhu (ZHu 2012) to estimate a *Z*-score statistic at each SNP position as a measure of the deviation in the local ancestries with respect to the average ancestry in the genome. This scan was conducted for the three ancestries separately, and an excess of locus-specific ancestry in a segment was considered significant if a large deviation was detected (i.e., *Z* score>|3|, *P* < 2.7×10^−3^). In those regions showing large ancestry deviations, genetic diversity was evaluated on the basis of the mean SNP heterozygosity estimates and the mean number of subjects with SNPs from those regions that were contained in ROH stretches. The statistical significance of the differences in these regions compared with data from the whole genome was assessed by Wilcoxon rank sum tests. Enrichment analyses were conducted for all regions with ancestry deviations together based on the peak regions defined by a *Z* score > 3 on hg19 using the Genomic Regions Enrichment of Annotations Tool (GREAT) (McLean et al. 2010). A hypergeometric test was used to estimate the significance of ontology term enrichment in the unique genes extracted in those regions with respect to the set of all genes in the genome. This analysis was evaluated for annotations in two particular ontologies (human diseases and MSigDB pathways), and the significance was corrected for multiple comparisons with a false discovery rate (FDR *q* value).

### Whole Genome Sequencing Data

Given that deviations of local ancestry often contain well-known targets of selection (i.e., *LCT* on chr2 and the HLA genes on chr6), we accessed data from deep WGS from a subset of 14 individuals (two per island) to further explore if the other regions showing deviations also harbored putative signals of selection. Briefly, DNA samples were processed with a Nextera DNA Prep kit with dual indexes following the manufacturer’s recommendations (Illumina Inc., San Diego, CA). Library sizes were checked on a TapeStation 4200 (Agilent Technologies, Santa Clara, CA). The concentration of each library was determined by a Qubit dsDNA HS Assay (Thermo Fisher, Waltham, MA). As a control, a PhiX DNA sample (1%) was also sequenced with the samples. Samples were sequenced to an average depth of 36.5× (range 24–45×) with paired-end 150-base reads on a HiSeq 4000 instrument (Illumina). Reads were preprocessed with bcl2fastq v2.18 and aligned to hg19 with BWA-MEM 0.7.15-r1140 (Li and Durbin 2010), and the BAMs were processed with Qualimap v2.2.1 (Okonechnikov et al. 2016), SAMtools v1.3 (Li et al. 2009), and Picard v2.1.1 (http://broadinstitute.github.io/picard). Variant calls were obtained with HaplotypeCaller in GATK (v3.7) (DePristo et al. 2011) following best practices workflow recommendations. These analyses were conducted in the Teide-HPC Supercomputing facility (http://teidehpc.iter.es/en). Sequencing data are available in http://www.iter.es/wp-content/uploads/2018/09/chr3_data_from_Canary_Islanders_MBE-Guillen-Guio-et-al.2018.vcf_.tar.gz for chromosome 3 and in http://www.iter.es/wp-content/uploads/2018/09/chr13_data_from_Canary_Islanders_MBE-Guillen-Guio-et-al.2018.vcf_.tar.gz for chromosome 13.

### Detection of Natural Selection

WGS data from Canary Islanders were used to extract the genotypes for the four described SNPs located upstream of *LCT* that are involved in lactase persistence as reported previously in the literature: rs4988235, rs41525747, rs41380347, and rs145946881 (Ingram et al. 2009). Furthermore, biallelic SNPs identified from WGS from the chr3 and chr13 regions defined by a *Z* score>|3| were used to ascertain the existence of candidate signals of evolutionary adaptation. To do so, we assessed Tajima’s *D* and PBS (Yi et al. 2010) in 10-kb windows, registering the observed local minima for Tajima’s *D* and the largest PBS scores for each chromosome region. Observed Tajima’s *D* and PBS statistics were compared against a null distribution generated from 5,000 neutral simulations under a simplified demographic model (details below). Statistical significance was calculated using Hudson’s “sample_stats” software (http://home.uchicago.edu/rhudson1/source/mksamples.html).

For the simulations, we used msHOT software (Hellenthal and Stephens 2007). As parameters, we used those from the reference model of Gutenkunst et al. (2009) for the general African (simulated population #1), European (#2) and Asian (#3) populations. These parameters were slightly modified to include a fourth population sample representing the Canary Islanders (#4). The output only included a set of 28 haplotypes representing the Canarian sample, equivalent in number to those assessed by WGS. To model the Canary Islanders’ demography, we assumed a simplified model that attempted to reflect, in a broad sense, their history according to historical records and genetically supported evidence of their effective population size (Ne) in pre-European times. No attempt was made to infer the most likely parameters for the Canary Islanders. In general terms, we assumed a small constant-sized isolated population of African origin, starting from 10,000 years BP, which suffered a strong decline in population size after European colonization of the Archipelago (simulated as an instant admixture occurring 500 years BP). The specific command line was as follows:

*./msHOT 28 5000 -t tbs -r tbs 10000 -I 4 0 0 0 28 -n 1 1.68202 -n 2 3.73683 -n 3 7.29205 -n 4 3.73683 -g 2 116.010723 -g 3 160.246047 -ma x 0.881098 0.561966 0 0.881098 x 2.79746 10 0.561966 2.79746 x 0 0 1 0 x -es 0.0005 4 0.1 -ej 0.0005 5 2 -en 0.0005 4 0.068 -ej 0.0135 4 1 -ej 0.028985 3 2 -en 0.028985 2 0.287184 -eM 0.028985 28 -ej 0.197963 2 1 -en 0.303501 1 1 < random_thetas-rhos.txt > output_file.ms*

The initial values of theta were inferred from the comparison of human and chimp orthologous sequences for each 10-kb region using a mutation rate based on the average number of substitutions per site (with the Jukes and Cantor correction) as assessed by DnaSP v5.1 (Librado and Rozas 2009). The rho parameter was estimated from the deCODE Genetics sex-averaged recombination-rate track in the UCSC Genome Browser (https://genome-euro.ucsc.edu). Uncertainty in the estimates of theta and rho was considered by generating a random and normally distributed set of values for these parameters, with mean equal to the estimated value and variance equal to the mean, from which we sampled a pair of values in each simulation.

To estimate the Ne of Canary Island aborigines in pre-European times, we accessed the publicly available Guanche genome (gun011; accession number ENA: PRJEB86458) with the largest depth-of-coverage (3.9×) from Rodríguez-Varela et al. (2017). These data correspond to a male found in Tenerife for which radiocarbon dating supports an age of 1,216 ± 27 years BP (the oldest one analyzed with the lowest contamination levels), which predates the European colonization of the Canary Islands. We used a total of 187.7 million reads mapped to the GRCh37 human reference genome as single-end reads. SAMtools (v1.3) and BCFtools (v1.3.1) were used to generate whole-genome consensus sequences at loci with a minimum mapping quality of 30 and a minimum and maximum read-depth of 3 and 20, respectively. Finally, the Pairwise Sequentially Markovian Coalescent (PSMC) method (Li and Durbin 2011) was used on the reads with base qualities >20 to estimate the Ne of the ancestral aboriginal population assuming 33 years per generation, a mutation rate of 2.5×10^−8^, and a range of background false negative rates (FNRs) of 0.0–0.3 for variant discovery. Considering the broad range of FNRs assumed, this analysis yielded a uniform Ne in the range of 470–560 for the Guanche population.

## Supplementary Material

Supplementary data are available at *Molecular Biology and Evolution* online.

## Supplementary Material

Supplementary Data
