# Supplementary material for: Genomic Analyses of Human European Diversity at the Southwestern Edge: Isolation, African Influence and Disease Associations in the Canary Islands
Source: Mol Biol Evol. 2018 Oct 5;35(12):3010–26. doi: 10.1093/molbev/msy190 (PMC6278859; doi:10.1093/molbev/msy190)
Supplement: Supplementary Data [file msy190_supp.zip › Supplementary_Figures_MBE_CFlores.pdf]

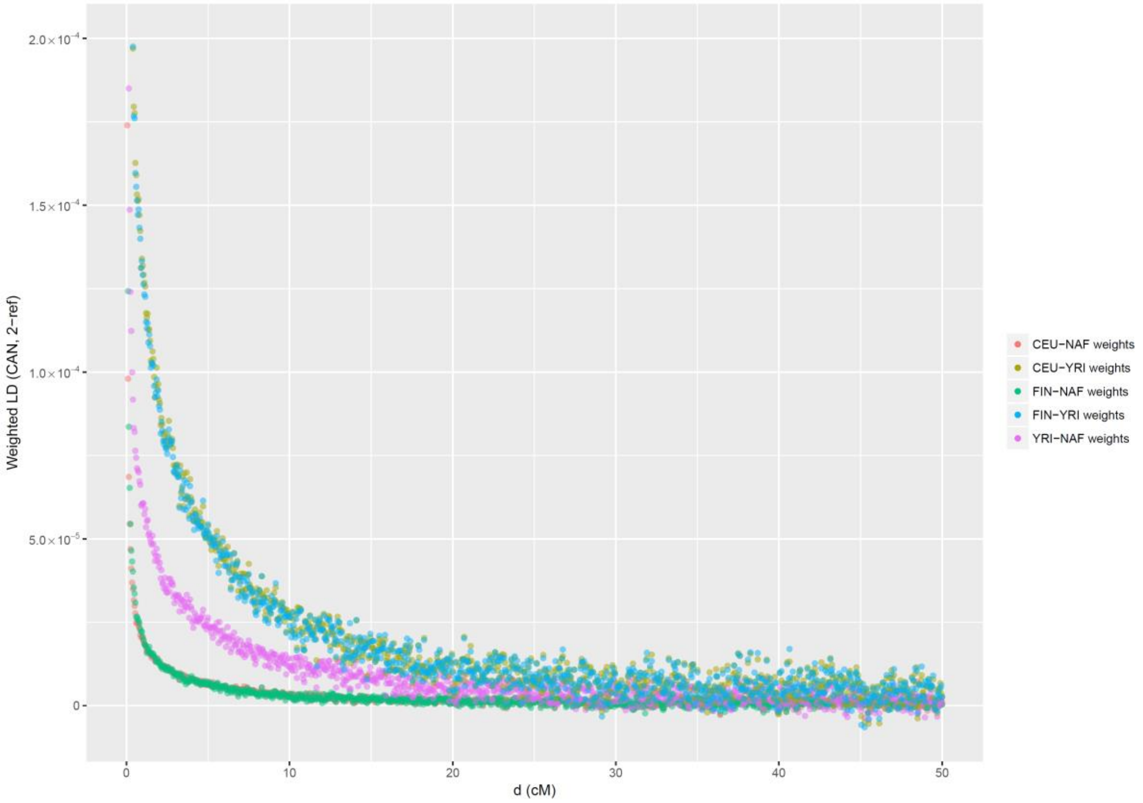

Supplementary Figure 1. ALDER results for 2-reference weighted LD computations in exemplar population pairs.

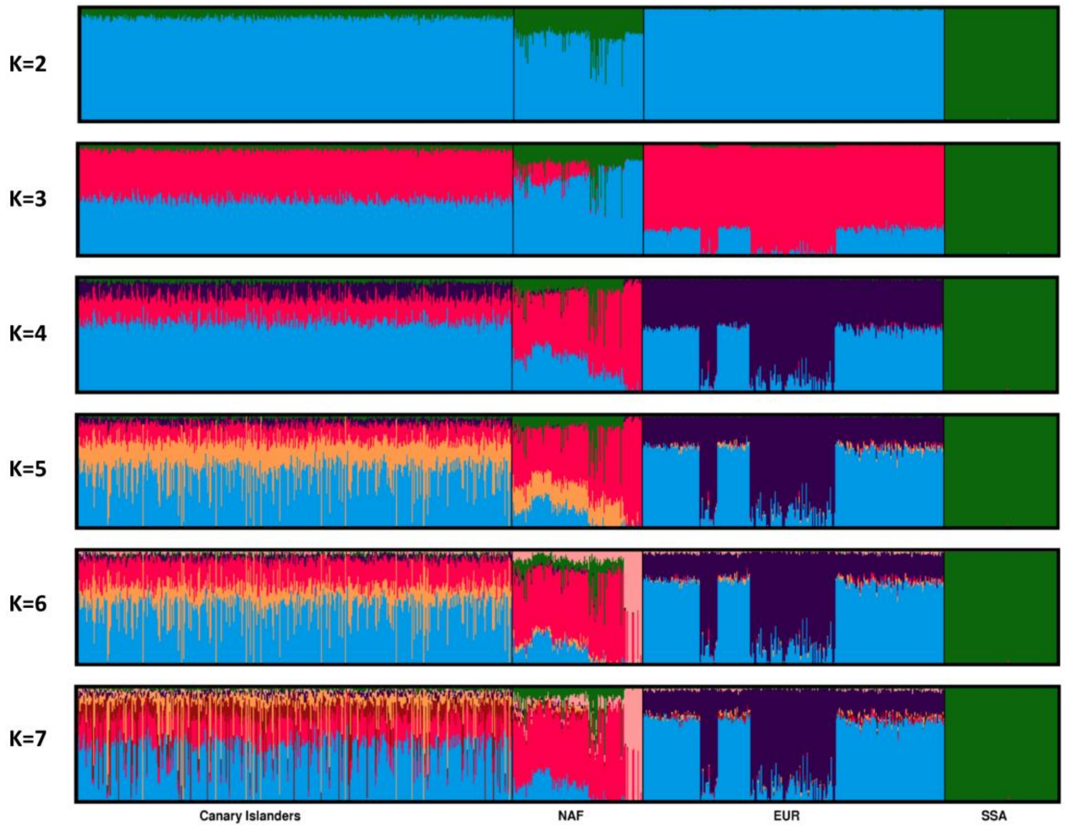

Supplementary Figure 2. ADMIXTURE results from K= 2 through 7. Individuals are represented as vertical lines, and each K ancestral genetic cluster is represented by a color. The lowest CV error was obtained at K=4, differentiating two ancestry clusters in Europeans. For K>5 a new genetic cluster arises mainly assigned to the Tunisian population, revealing novel ancestry clusters in populations other than SSA.

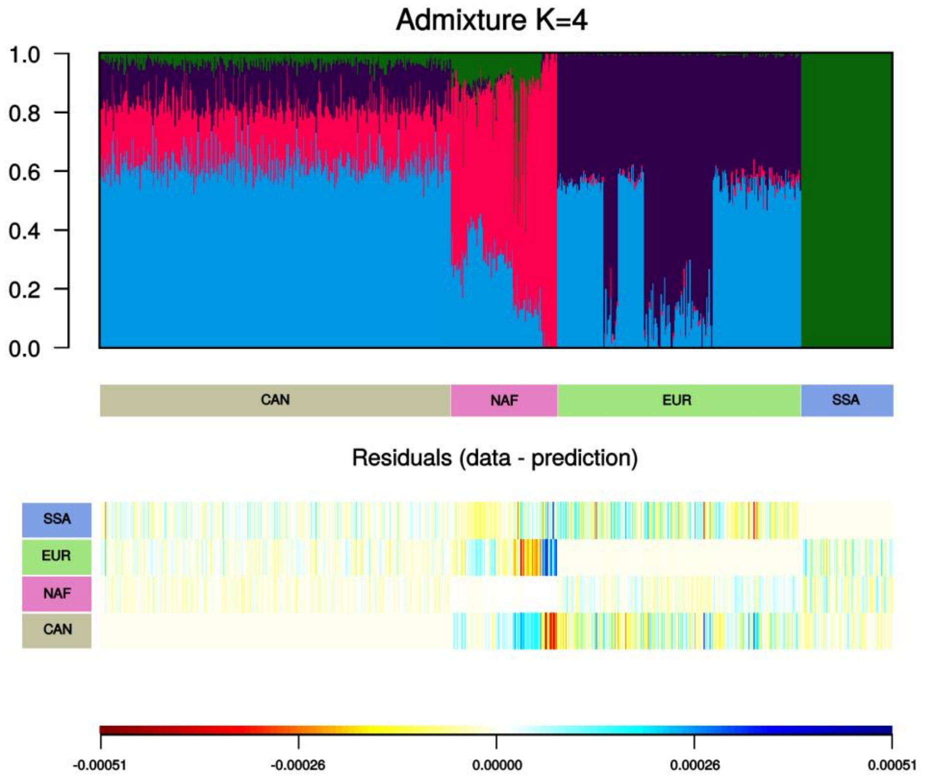

Supplementary Figure 3. Admixture model fitting as provided by badMIXTURE. Upper panel: Ancestry clusters as estimated by ADMIXTURE (K=4). Lower panel: Residuals from the goodness of fit of the model with CHROMOPAINTER measures of haplotype sharing with individuals from NAF, EUR and SSA groups.

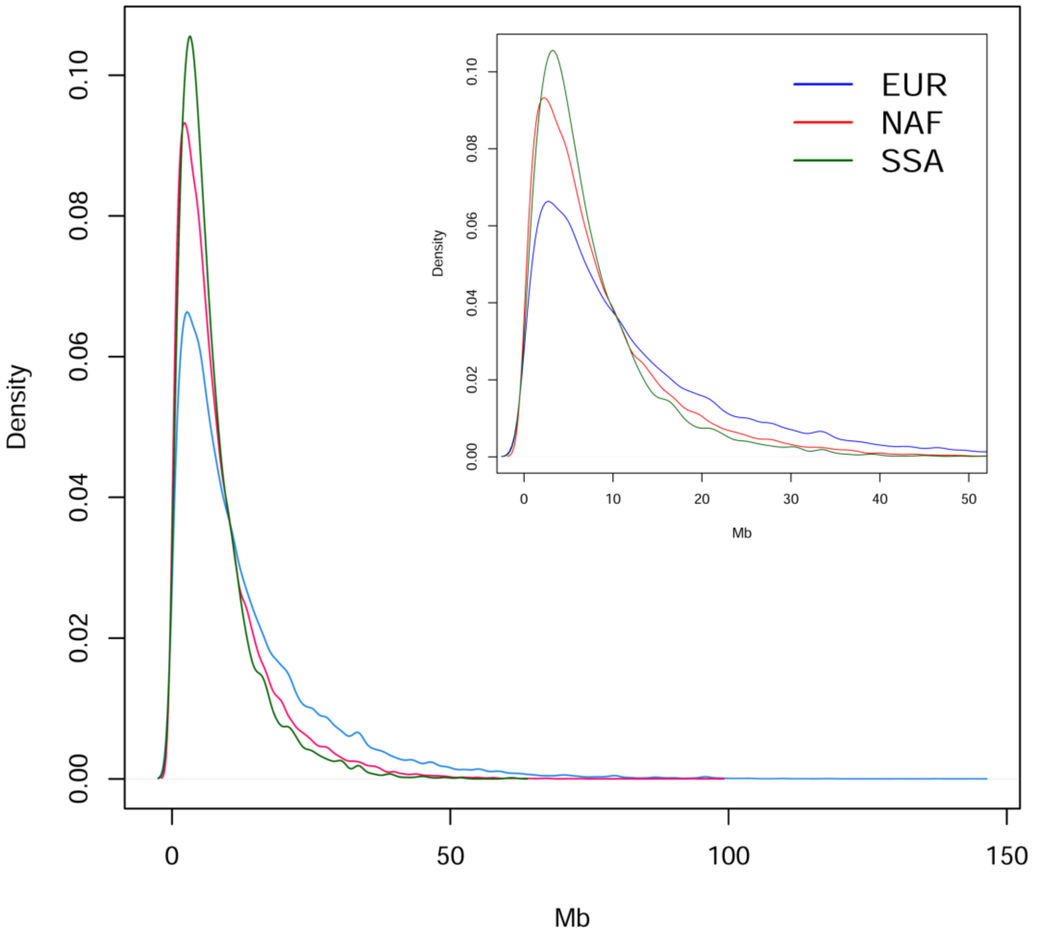

Supplementary Figure 4. Density plot of approximate ELAI block size estimates.

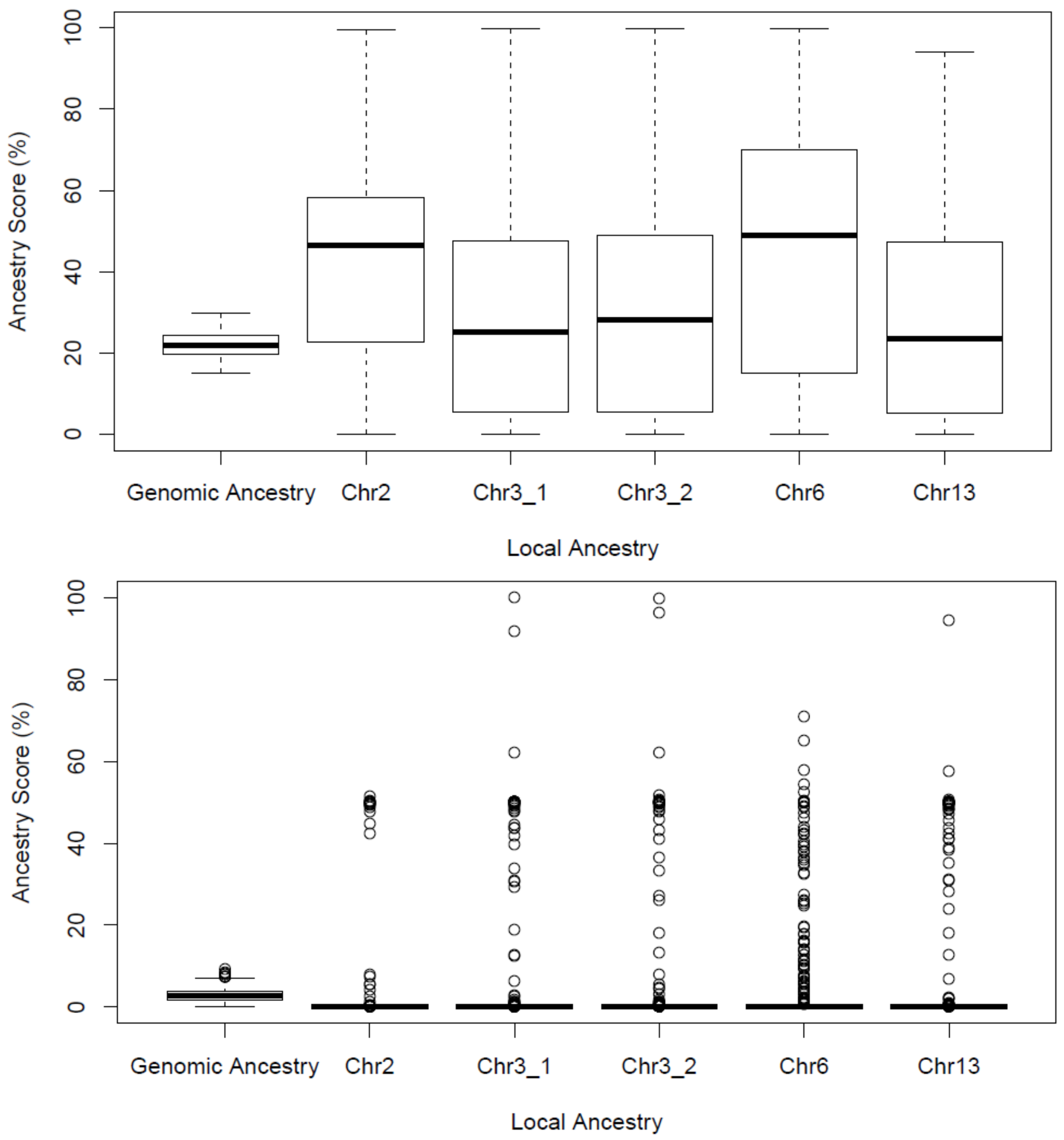

Supplementary Figure 5. Regional plots of ancestry scores for the regions with large deviations in NAF (top) and SSA (bottom) ancestries. Chr3_1 and Chr3_2 correspond to chr3:10,539,482-11,710,471 and chr3:177,443,968-178,679,751, respectively.

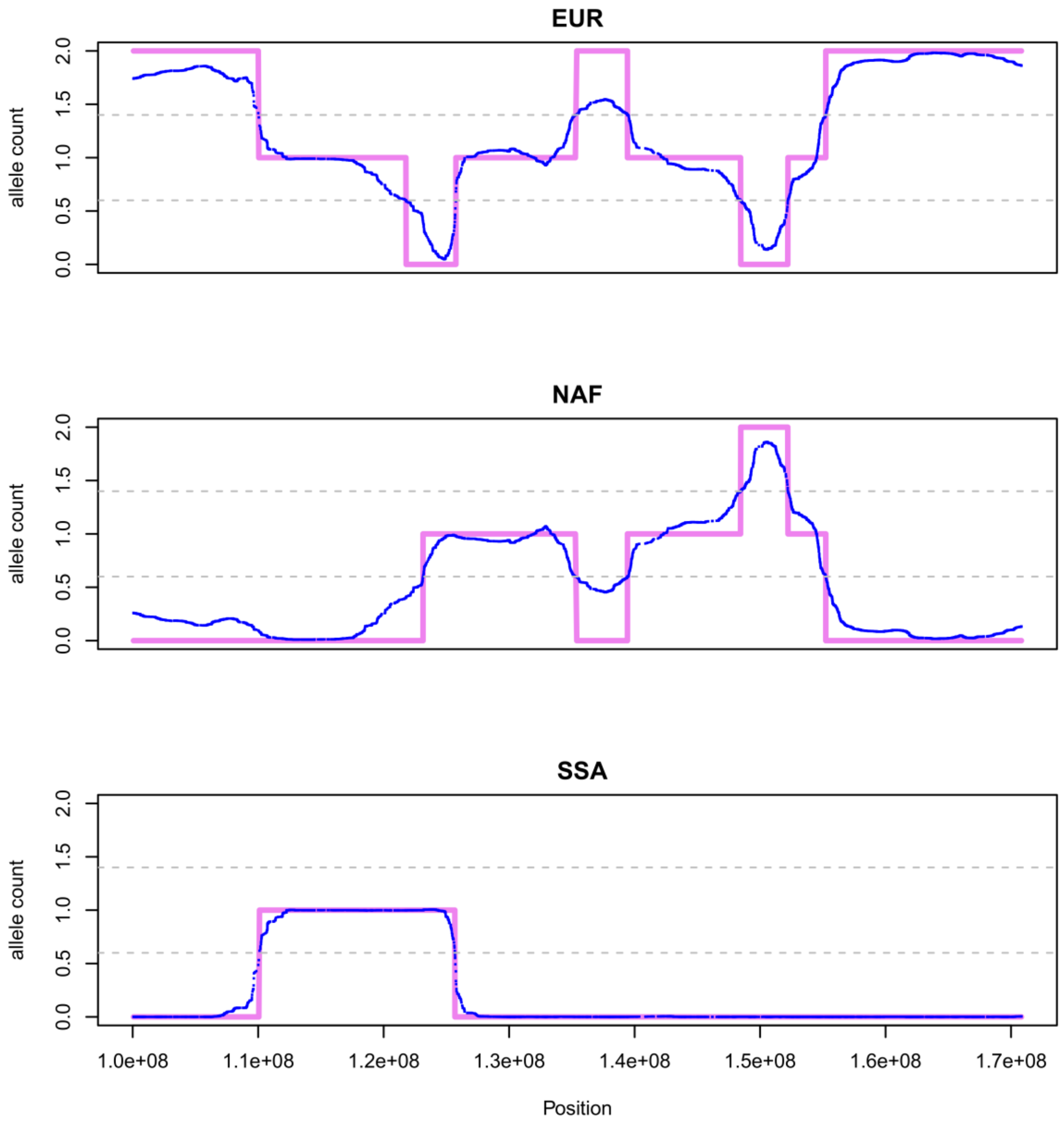

Supplementary Figure 6. Inference of local ancestry by ELAI. The plot shows an example of inference in a chromosome region (one panel of each parental population) comparing ELAI allele dosages (blue) with the approximations to the next non-negative integer (pink) to assist in the estimation of ELAI block lengths.
