# Supplementary material for: Genomic Analyses of Human European Diversity at the Southwestern Edge: Isolation, African Influence and Disease Associations in the Canary Islands
Source: Mol Biol Evol. 2018 Oct 5;35(12):3010–26. doi: 10.1093/molbev/msy190 (PMC6278859; doi:10.1093/molbev/msy190)
Supplement: Supplementary Data [file msy190_supp.zip › Supplementary_Tables_MBE_CFlores.pdf]

Supplementary Table 1. Genes mapping to the regions with large deviations in ancestry.

ABCF1, ABHD16A, ABT1, ACMSD, ACOT13, AGER, AGPAT1, AIF1, ALDH5A1, ANKS1A, APOM, ARHGAP15, ARMC12, ATAT1, ATF6B, ATG7, ATP2B2, ATP6V1G2, B3GALT4, BAG6, BAK1, BRD2, BRPF3, BTN1A1, BTN2A1, BTN2A2, BTN3A1, BTN3A2, BTN3A3, BTNL2, C2, C4A, C4B_2, C6orf1, C6orf10, C6orf106, C6orf136, C6orf15, C6orf222, C6orf25, C6orf47, C6orf48, C6orf62, CCHCR1, CCNT2, CDKN1A, CDSN, CFB, CLIC1, CLPS, CLPSL1, CLPSL2, COL11A2, CSNK2B, CUTA, CXCR4, CYP21A2, DARS, DAXX, DCDC2, DDAH2, DDR1, DDX39B, DEF6, DHX16, DIAPH3, DPCR1, EGFL8, EHMT2, ETV7, FAM65B, FANCE, FKBP5, FKBPL, FLOT1, GABBR1, GMNN, GNL1, GPANK1, GPLD1, GPSM3, GPX5, GPX6, GRM4, GTF2H4, HFE, HIST1H1A, HIST1H1B, HIST1H1C, HIST1H1D, HIST1H1E, HIST1H1T, HIST1H2AA, HIST1H2AB, HIST1H2AC, HIST1H2AD, HIST1H2AE, HIST1H2AG, HIST1H2AH, HIST1H2AJ, HIST1H2AK, HIST1H2AL, HIST1H2AM, HIST1H2BA, HIST1H2BB, HIST1H2BC, HIST1H2BD, HIST1H2BE, HIST1H2BF, HIST1H2BG, HIST1H2BH, HIST1H2BI, HIST1H2BJ, HIST1H2BK, HIST1H2BL, HIST1H2BM, HIST1H2BN, HIST1H2BO, HIST1H3A, HIST1H3B, HIST1H3C, HIST1H3D, HIST1H3E, HIST1H3F, HIST1H3G, HIST1H3H, HIST1H3I, HIST1H3J, HIST1H4A, HIST1H4B, HIST1H4C, HIST1H4D, HIST1H4E, HIST1H4F, HIST1H4G, HIST1H4H, HIST1H4I, HIST1H4J, HIST1H4K, HIST1H4L, HLA-A, HLA-B, HLA-C, HLA-DMA, HLA-DMB, HLA-DOA, HLA-DOB, HLA-DPA1, HLA-DPB1, HLA-DQA1, HLA-DQA2, HLA-DQB1, HLA-DQB2, HLA-DRA, HLA-DRB1, HLA-DRB5, HLA-E, HLA-F, HLA-G, HMGA1, HMGN4, HNMT, HRH1, HSD17B8, HSPA1A, HSPA1B, HSPA1L, IER3, IP6K3, ITPR3, KAAG1, KCNMB2, KCTD20, KIAA0319, KIFC1, KYNU, LCT, LEMD2, LHFPL5, LRP1B, LRRC16A, LSM2, LST1, LTA, LTB, LY6G5B, LY6G5C, LY6G6C, LY6G6D, LY6G6F, MAP3K19, MAPK13, MAPK14, MAS1L, MCCD1, MCM6, MDC1, MGAT5, MICA, MICB, MLN, MOG, MRPS18B, MRS2, MUC21, MUC22, NCKAP5, NCR3, NELFE, NEU1, NFKBIL1, NKAPL, NOTCH4, NRM, NRSN1, NUDT3, NXPH2, OR10C1, OR11A1, OR12D2, OR12D3, OR14J1, OR2B2, OR2B3, OR2B6, OR2H1, OR2H2, OR2J2, OR2J3, OR2W1, OR5V1, PACSIN1, PBX2, PCDH17, PCDH20, PCDH9, PFDN6, PGBD1, PHF1, PNPLA1, POM121L2, POU5F1, PPARD, PPP1R10, PPP1R11, PPP1R18, PPT2, PRR3, PRRC2A, PRRT1, PRSS16, PSMB8, PSMB9, PSORS1C1, PSORS1C2, PXT1, R3HDM1, RAB3GAP1, RGL2, RING1, RNF39, RNF5, RPL10A, RPP21, RPS10, RPS18, RXRB, SCGN, SCUBE3, SFTA2, SKIV2L, SLC17A1, SLC17A2, SLC17A3, SLC17A4, SLC26A8, SLC39A7, SLC44A4, SLC6A1, SLC6A11, SNRPC, SPDEF, SPOPL, SRPK1, SRSF3, STK19, STK38, SYNGAP1, TAF11, TAP1, TAP2, TAPBP, TCF19, TCP11, TDP2, TDRD3, TEAD3, THSD7B, TMEM163, TNF, TNXB, TRIM10, TRIM15, TRIM26, TRIM27, TRIM31, TRIM38, TRIM39, TRIM39-RPP21, TRIM40, TUBB, TULP1, UBD, UBXN4, UHRF1BP1, VARS, VARS2, VGLL4, VPS52, VWA7, WDR46, ZBTB12, ZBTB22, ZBTB9, ZFP57, ZKSCAN3, ZKSCAN4, ZKSCAN8, ZNF165, ZNF184, ZNF311, ZNF322, ZNF391, ZNF76, ZNRD1, ZRANB3, ZSCAN16, ZSCAN23, ZSCAN26, ZSCAN31, ZSCAN9

Supplementary Table 2. Significantly enriched human diseases in regions with large deviations in ancestry.

Term	q-value	Fraction in annotation
Psoriasis	3.44E-10	7.18%
Mucocutaneous lymph node syndrome	2.13E-08	3.74%
Vascular skin disease	2.88E-08	5.75%
Lymphadenitis	4.16E-08	3.74%
Integumentary system disease	1.03E-07	11.21%
Skin disease	1.10E-07	10.92%
Peripheral vascular disease	5.50E-07	6.03%
Polyarteritis nodosa	6.91E-07	3.74%
Nephrotic syndrome	8.71E-06	3.16%
Nephrosis	1.37E-05	3.16%
Graves' disease	3.56E-05	3.16%
Sarcoidosis	4.96E-05	3.16%
Autoimmune disease	7.32E-05	10.92%
Hyperthyroidism	2.33E-04	3.16%
Thyrotoxicosis	2.44E-04	3.16%
Hepatitis	3.08E-04	6.90%
Goiter	4.04E-04	3.16%
Autoimmune disease of endocrine system	4.38E-04	3.16%
Cytomegalovirus infectious disease	4.53E-04	3.45%
(+)ssRNA virus infectious disease	4.56E-04	5.46%
Psoriatic arthritis	4.65E-04	2.01%
Pemphigus	4.65E-04	2.01%
Allergy	4.66E-04	4.31%
Duodenal ulcer	4.81E-04	1.72%
Cystic echinococcosis	5.30E-04	0.86%
Leprosy	5.44E-04	2.01%
Flaviviridae infectious disease	5.65E-04	4.89%
Echinococcosis	6.07E-04	1.15%
Demyelinating disease	6.73E-04	5.46%
Behcet's disease	6.78E-04	2.59%
Hepatitis C	7.20E-04	4.60%
DNA virus infectious disease	9.47E-04	7.76%
Intrahepatic cholestasis	1.04E-03	1.44%
Multiple sclerosis	1.04E-03	5.17%
Demyelinating disease of central nervous system	1.22E-03	5.17%
Hepatitis B	1.24E-03	4.02%
Pulmonary tuberculosis	1.30E-03	2.01%
Cavernous hemangioma	1.36E-03	0.86%
Liver disease	1.84E-03	8.05%
Congenital adrenal hyperplasia	1.84E-03	1.15%
Primary bacterial infectious disease	1.86E-03	4.60%
Arthritis	1.89E-03	8.05%
Bone inflammation disease	1.91E-03	8.33%
Dengue disease	2.20E-03	1.44%
Rheumatic fever	2.20E-03	1.44%
Urticaria	2.41E-03	2.01%
Food allergy	2.60E-03	2.59%
Glucose metabolism disease	3.26E-03	10.06%
Diabetes mellitus	3.26E-03	9.77%
Peptic ulcer	3.53E-03	2.01%
Nasopharynx carcinoma	3.77E-03	3.74%
Viral infectious disease	4.04E-03	10.06%
Brucellosis	4.31E-03	1.72%
Chagas cardiomyopathy	4.48E-03	0.86%
Neuromyelitis optica	5.01E-03	1.15%
Parasitic infectious disease	5.06E-03	3.16%
Systemic lupus erythematosus	5.14E-03	4.60%
Severe acute respiratory syndrome	5.67E-03	1.72%
Bacterial infectious disease	5.82E-03	4.89%
RNA virus infectious disease	5.89E-03	6.32%
Rheumatoid arthritis	6.17E-03	6.32%
Nidovirales infectious disease	6.18E-03	1.72%
Vitiligo	6.32E-03	2.01%

Prostatitis	6.41E-03	0.86%
Celiac disease	6.83E-03	2.30%
Hepatobiliary disease	7.70E-03	8.33%
Aplastic anemia	7.83E-03	2.01%
Bullous skin disease	7.83E-03	2.01%
Herpesviridae infectious disease	8.06E-03	4.02%
Vasculitis	8.27E-03	2.87%
Purpura	8.88E-03	2.01%
Pure red-cell aplasia	8.92E-03	0.57%
Alcoholic pancreatitis	9.01E-03	0.86%
Autoimmune disease of gastrointestinal tract	9.07E-03	3.45%
Mycobacterium infectious disease	1.03E-02	3.16%
Hypotrichosis	1.07E-02	1.72%
Adrenal hyperplasia	1.15E-02	1.15%
Parasitic helminthiasis infectious disease	1.16E-02	1.44%
Gastrointestinal system disease	1.18E-02	14.66%
Dengue shock syndrome	1.19E-02	0.86%
Primary Actinomycetales infectious disease	1.28E-02	3.16%
Lupus erythematosus	1.30E-02	4.89%
Autoimmune disease of the nervous system	1.33E-02	1.72%
Dermatitis	1.40E-02	4.31%
Hair disease	1.43E-02	1.72%
Upper respiratory tract disease	1.67E-02	3.45%
Open-angle glaucoma	1.83E-02	1.72%
Myasthenia gravis	1.89E-02	1.44%
Neuromuscular junction disease	2.05E-02	1.44%
Nose disease	2.06E-02	2.30%
Facial neoplasm	2.10E-02	0.57%
Bacterial prostatitis	2.10E-02	0.57%
Spondylitis	2.64E-02	1.72%
Ankylosing spondylitis	2.64E-02	1.72%
Flavivirus infectious disease	2.74E-02	1.44%
Arthropathy	2.77E-02	2.01%
Primitive neuroectodermal tumor	2.77E-02	6.03%
Alopecia	2.94E-02	1.44%
Sickle cell anemia	2.96E-02	1.15%
Pancreatitis	3.03E-02	2.30%
Diabetes mellitus type 1	3.03E-02	0.86%
Uveal disease	3.17E-02	1.44%
Uveitis	3.27E-02	1.15%
Vascular hemostatic disease	3.35E-02	3.74%
dsDNA virus infectious disease	3.37E-02	5.17%
Embryonal cancer	3.44E-02	6.03%
Collagen disease	3.47E-02	2.87%
Sjogren's syndrome	3.47E-02	1.72%
Rubella	3.48E-02	0.86%
Dengue hemorrhagic fever	3.48E-02	0.86%
Alloimmunization	3.49E-02	0.57%
Alcoholic fatty liver	3.49E-02	0.57%
Complex regional pain syndrome	3.49E-02	0.57%
Crohn's disease	3.50E-02	2.87%
Tuberculosis	3.72E-02	2.59%
Nephritis	3.73E-02	3.16%
Spondyloarthropathy	4.28E-02	1.72%
Allergic rhinitis	4.33E-02	2.01%
Retinal degeneration	4.57E-02	3.45%
Rhinitis	4.78E-02	2.01%

Supplementary Table 3. Significantly enriched MSigDB pathways in regions with large deviations in ancestry.

Term	q-value	Fraction in annotation
Systemic lupus erythematosus	6.79E-72	18.39%
Genes involved in RNA Polymerase I Promoter Opening	1.42E-54	11.78%
Genes involved in RNA Polymerase I Transcription	2.35E-49	12.36%
Genes involved in Meiotic Recombination	5.36E-48	12.07%
Genes involved in Amyloids	3.20E-47	11.78%
Genes involved in RNA Polymerase I, RNA Polymerase III, and Mitochondrial Transcription	1.06E-41	12.36%
Genes involved in Meiosis	2.10E-41	12.07%
Genes involved in Packaging Of Telomere Ends	9.47E-40	8.91%
Genes involved in Transcription	3.06E-35	13.51%
Genes involved in Deposition of New CENPA-containing Nucleosomes at the Centromere	6.53E-35	8.91%
Genes involved in Meiotic Synapsis	1.38E-32	8.91%
Genes involved in Telomere Maintenance	1.06E-31	8.91%
Genes involved in Chromosome Maintenance	1.11E-24	8.91%
Antigen processing and presentation	1.83E-20	6.90%
Type I diabetes	2.36E-20	5.46%
Allograft rejection	2.45E-20	5.17%
Graft-versus-host disease	8.67E-20	5.17%
Autoimmune thyroid disease	1.95E-15	4.89%
Genes involved in Cell Cycle	3.57E-14	10.92%
Viral myocarditis	5.83E-13	4.89%
Asthma	3.19E-12	3.45%
Intestinal immune network for IgA production	2.14E-09	3.45%
Leishmania infection	2.59E-09	4.02%
Cell adhesion molecules (CAMs)	2.98E-08	4.89%
Genes involved in Antigen Presentation: Folding, assembly and peptide loading of class I MHC	9.61E-08	2.30%
Genes involved in Interferon gamma signaling	8.79E-07	3.16%
Genes involved in Translocation of ZAP-70 to Immunological synapse	3.69E-06	1.72%
Genes involved in Endosomal/Vacuolar pathway	6.53E-06	1.44%
Genes involved in Phosphorylation of CD3 and TCR zeta chains	9.71E-06	1.72%
Genes involved in PD-1 signaling	2.25E-05	1.72%
Genes involved in ER-Phagosome pathway	8.82E-05	2.59%
Genes involved in Apoptosis induced DNA fragmentation	1.21E-04	1.44%
Genes involved in Generation of second messenger molecules	3.28E-04	1.72%
Genes involved in MHC class II antigen presentation	3.91E-04	2.87%
Genes involved in Antigen processing-Cross presentation	4.61E-04	2.59%
Genes involved in Interferon Signaling	1.62E-03	3.45%
Genes involved in Downstream TCR signaling	2.05E-03	1.72%
Complement Pathway	1.42E-02	1.15%
Genes involved in Adaptive Immune System	1.57E-02	6.32%
Genes involved in TCR signaling	1.70E-02	1.72%
Natural killer cell mediated cytotoxicity	3.32E-02	2.59%
Genes involved in Costimulation by the CD28 family	3.44E-02	1.72%
Genes involved in Interferon alpha/beta signaling	3.99E-02	1.72%
Lectin Induced Complement Pathway	4.03E-02	0.86%
